# Altered motor, anxiety-related and attentional task performance at baseline associate with multiple gene copies of the vesicular acetylcholine transporter and related protein overexpression in ChAT::Cre+ rats

**DOI:** 10.1007/s00429-019-01957-y

**Published:** 2019-09-10

**Authors:** Craig P. Mantanona, Johan Alsiö, Joanna L. Elson, Beth M. Fisher, Jeffrey W. Dalley, Timothy Bussey, Ilse S. Pienaar

**Affiliations:** 1 Faculty of Health and Life Sciences, Northumbria University, Newcastle upon Tyne, UK; 2 Department of Psychology, The Behavioral and Clinical Neuroscience Institute, University of Cambridge, Downing Street, Cambridge, UK; 3 Institute of Genetic Medicine, Newcastle University, Newcastle upon Tyne, UK; 4 Department of Physiology and Pharmacology, Robarts Research Institute, University of Western Ontario, London, ON Canada; 5 School of Life Sciences, University of Sussex, Falmer, BN1 9PH UK

**Keywords:** ChAT::Cre rats, Cre-recombinase, 5-CSRTT, HPA endocrine axis, Touchscreen-based behavioral testing, Vesicular acetylcholine transporter

## Abstract

Transgenic rodents expressing Cre recombinase cell specifically are used for exploring mechanisms regulating behavior, including those mediated by cholinergic signaling. However, it was recently reported that transgenic mice overexpressing a bacterial artificial chromosome containing *choline acetyltransferase* (*ChAT*) gene, for synthesizing the neurotransmitter acetylcholine, present with multiple *vesicular acetylcholine transporter* (*VAChT*) gene copies, resulting in altered cholinergic tone and accompanying behavioral abnormalities. Since ChAT::Cre+ rats, used increasingly for understanding the biological basis of CNS disorders, utilize the mouse ChAT promotor to control Cre recombinase expression, we assessed for similar genotypical and phenotypical differences in such rats compared to wild-type siblings. The rats were assessed for mouse *VAChT* copy number, VAChT protein expression levels and for sustained attention, response control and anxiety. Rats were also subjected to a contextual fear conditioning paradigm using an unconditional fear-inducing stimulus (electrical foot shocks), with blood samples taken at baseline, the fear acquisition phase and retention testing, for measuring blood plasma markers of hypothalamic–pituitary–adrenal gland (HPA)-axis activity. ChAT::Cre+ rats expressed multiple mouse *VAChT* gene copies, resulting in significantly higher VAChT protein expression, revealed anxiolytic behavior, hyperlocomotion and deficits in tasks requiring sustained attention. The HPA-axis was intact, with unaltered circulatory levels of acute stress-induced corticosterone, leptin and glucose. Our findings, therefore, reveal that in ChAT::Cre+ rats, *VAChT* overexpression associates with significant alterations of certain cognitive, motor and affective functions. Although highly useful as an experimental tool, it is essential to consider the potential effects of altered cholinergic transmission on baseline behavior in ChAT::Cre rats.

## Introduction

Central cholinergic signaling is required for regulating aspects of memory, motivation and mood. On the other hand, cholinergic deficiency contributes to the neuropsychiatric symptoms associated with Alzheimer’s disease (AD), with cholinomimetic therapies that may ameliorate such symptoms (Felder et al. 2018). In addition, although the main motor deficits characterizing Parkinson’s disease (PD) patients stem from the near total loss of midbrain dopaminergic neurons, the concomitant loss and/or dysfunction of cholinergic neurons is believed to underlie such patients’ cognitive decline (Perez-Lloret and Barrantes 2016; Bury et al. 2017). Hyperactivity of brain cholinergic systems could also underlie the cognitive disturbances observed in patients with affective disorders (Saricicek et al. 2012; Mineur et al. 2013), while additional evidence for this derives from clinical and preclinical studies which revealed that cholinergic receptor blockers can induce antidepressant-like responses (Furey and Drevets 2006). Furthermore, increasing data from clinical pharmacology, neuroimaging and postmortem studies implicates the cholinergic system in the pathogenesis of schizophrenia (Sarter et al. 2012). Conversely, growing evidence suggest that pharmacologically increasing cholinergic signaling could improve several symptom clusters, including visual hallucinations, in schizophrenia patients (Foster et al. 2012). However, despite the accumulating evidence in support of cholinergic perturbations as an etiological factor in several neuropsychiatric human diseases, patients remain mostly unresponsive to current cholinergic-based pharmacological therapies. This highlights a need for establishing appropriate mammalian models for understanding the cholinergic roots of these disorders and for in vivo validation of candidate therapeutics that are potentially more effective treatments, by specifically correcting the relevant pathophysiological mechanisms.

For modeling human disease, rats offer several distinct advantages over mice and other organisms. Benefits include increased spatial resolution due to rats’ larger sized heads, which provides practical advantages for performing surgical procedures, including intracerebral cannula implantation for localized drug infusion into a specific brain region, to determine the role of this region in a particular behavioral phenotype (Kokare et al. 2011). Proportionally, larger brain substructure sizes also lends itself to more precise implantation of microdialysis probes for sampling neurotransmitter concentrations, as the cannula causes less damage by affecting smaller regions. Although mice continue to offer distinct advantages over rats for precise targeting of gene expression to defined neuronal populations (Taniguchi et al. 2011; Madisen et al. 2012; Gerfen et al. 2013), tremendous strides made in developing a toolbox of transgenic rat lines have allowed for unprecedented interrogation of cell type-specific neural populations and -circuits. Such experimental strides are permitted by engineering cell-specific targeting in a Cre recombinase-dependent manner, along with advances made in engineered viral vectors. These transgenic rat technologies can be combined with opsin- and Designer Receptors Exclusively Activated by Designer Drugs (DREADDs), for untangling the neural substrates of a wide plethora of behaviors; these are beginning to be tapped in research studies (e.g., Sharma and Pienaar 2014, 2018).

Choline acetyltransferase (ChAT)::Cre+ transgenic rats, utilizing bacterial artificial chromosome (BAC) recombineering for introducing a transgene of interest under control of cholinergic neuronal-specific promoter elements (Witten et al. 2011) were generated to genetically restrict expression of Cre recombinase in cholinergic neurons. As an additional genetic element, the BAC is inserted within the genome, sometimes in multiple copies, with the original ChAT locus that remains intact.

Such rats are extremely useful in promoting understanding of cholinergic circuits (Aldrin-Kirk et al. 2018; Dautan et al. 2014, 2016; Gielow and Záborszky 2017) including their role in motor function and reward-related behaviors during both homeostatic and disease processes (Pienaar et al. 2015; Dautan et al. 2016; Xiao et al. 2016). The generation of these transgenic rats followed on from work using transgenic mice was designed to encode channelrhodopsin-2 (ChR2) protein under the control of the ChAT promoter (Heintz 2001). This design largely ignores the tandem gene arrangement for the cholinergic gene locus (Prado et al. 2017), where the gene for the vesicular acetylcholine transporter is nested within the first intron of the *ChAT* gene. Evidence is accumulating that in ChAT::Cre+ rodents, altered expression of the gene encoding for the vesicular acetylcholine transporter (VAChT), which serves as a key molecular component of acetylcholine (ACh) release by loading ACh molecules into the synaptic vesicles of cholinergic synapses, could modify baseline cholinergic tone. This modification could provide a mechanistic basis for changing aspects such as locomotion, operant conditioning, spatial and working memory, attention, as well as intravenous nicotine self-administration behaviors, as had been reported for ChAT-ChR2-eYFP BAC transgenic mice, compared to wild-type (Wt/Wt) and hemizygous (Tg/Wt) littermates (Kolisnyk et al. 2013; Sugita et al. 2016; Chen et al. 2018; Janickova et al. 2017).

Here, we determined the mRNA and protein expression levels of VAChT in ChAT::Cre+ rats compared to Wt (ChAT::Cre−) ones, to gain mechanistic understanding as to the influence exerted by altered expression of cholinergic signaling factors in performance in the five-choice serial reaction time task (5-CSRTT), a well-validated measure of attention and impulsivity (Robbins 2002). We further conducted motor and anxiety profiling in the transgenic rats compared to their Wt littermates, using a range of well-validated behavior tests. Due to compelling evidence that the cholinergic system plays a role in stress and, specifically in hypothalamic–pituitary–adrenal gland (HPA)-axis activation and regulation, we also determined whether ChAT::Cre+ rats manifest a dysregulation of this endocrine control system (Paul et al. 2015).

## Materials and methods

### Animals

The animal experiments were approved by an ethics panel at Northumbria University (Ref: BMS39UNNGB2015) and were performed in accordance with the Animals (Scientific Procedures) Act, 1986 (UK) for the care and use of experimental animals as well as the European Communities Council Directive (2010/63/EEC). All rats were examined weekly by a veterinarian and constantly monitored by experienced animal technicians for signs of pain or distress. Long-Evans Wt rats [Charles River, Kent, UK; Research Resource Identifier (RRID): RGD_2308852] were bred with hemizygous Long-Evans ChAT::Cre rats (Missouri Mutant Mouse Regional Resource Centre, University of Missouri, USA; RRID: RGD_10401204); both providers supplied up-to-date authentication reports. Once weaned, ear clippings were taken from each pup and analyzed for the *Cre recombinase* gene using polymerase chain reaction (PCR). The primers used were as described by Witten et al. (2011) and consisted of AAGAACCTGATGGACATGTTCAGGGATCG (forward) and CCACCGTCAGTACGTGAGATATCTTTAACC (reverse), which produced a positive band corresponding to 600 base pairs. The PCR cycling conditions were similar to those described by Witten et al. (2011).

Only mature male rats (weight: 270–310 g) were used in the experiments to minimize gender and hormonal influences on behavior (Keeley et al. 2015). As an additional control condition for analyzing mouse *VAChT* gene copy number, brain tissue was also collected from male littermate Wt control mice (weight: 25–30 g, on the C57BL/6 background, received from Charles River, UK).

Animals were housed in groups of 3–4 per cage in polypropylene cages measuring 56 × 38 × 22 cm (RC2F; North Kent Plastics Cages, Coalville, UK) within an animal care facility at the University of Cambridge, in a temperature (23 °C) and humidity (45–65%) controlled room with a reversed light/dark cycle (lights off between 7:00 and 19:00 h). The behavioral experiments were carried out at the same time each day, during the dark period which represents the rats’ nocturnal phase of the circadian cycle, during which they experience their most active, inquisitive and responsive period (Gorka and Adamik 1993). As the rats were maintained on a reverse light/dark cycle, this enabled us to conveniently test the rats during their activity phase.

One group was used for testing in the 5-CSRTT (ChAT::Cre+, *n* = 13; ChAT::Cre−, *n* = 8), whilst the second group was subjected to anxiety-profiling tasks, consisting of the open field (OF) arena, elevated plus maze (EPM), and light/dark box (LDB) (ChAT::Cre+, *n* = 8; ChAT::Cre−, *n* = 10). Finally, some of the rats (that had been subjected to either the 5-CSRTT or anxiety testing) were subjected to fear conditioning testing followed by blood/tissue analysis (ChAT::Cre+, *n* = 5; ChAT::Cre−, *n* = 8). During 5-CSRTT training, the animals had their weight monitored weekly and maintained at 85–95% of their free feeding body weight. During this period, the animals received 18 g of laboratory pellet chow once a day with ad libitum access to water. Animals used for anxiety profile tasks and fear conditioning followed by blood/tissue analysis had ad libitum access to food and water. OF, EPM, LDB, fear conditioning testing and blood sampling occurred between 14:00 and 17:00. The order of behavioral assessment was anxiety profile tasks (OF, EPM and LDB) followed by the contextual fear conditioning task (CFCT) and then blood sampling. No sample calculation was performed to estimate the number of rats used in each experimental group.

### Behavioral procedures

Blind scoring of the rats’ behavioral test performance was performed using automated computer software. Additionally, the experimenter was blind to genotype during rat-derived sample processing. For each behavioral test, rats were tested in a counterbalanced order with regard to genotype. Following testing of each animal, the apparatus was thoroughly cleaned with 70% ethanol (EtOH) to eliminate the residual odors and traces of the previously tested animal.

#### Open field testing

All behavioral procedures were conducted in a dark (visibility was 5 m), soundproofed room. For measuring behavior in the OF arena, rats were placed in the center of a circular field of 900 cm (diameter) × 38.5 cm (height). The apparatus consisted of an inner field (625 cm in diameter) surrounded by an outer field (375 cm from inner field edge to field’s edge). Room temperature (RT) and lighting were identical to that used for testing in the EPM (see below). Each rat was recorded for 5 min using a Yi Action Camera (Yi Technology, Seattle, USA) connected to a personal computer, with subsequent videos analyzed using ANY-maze software (Stoelting ANY-maze, Wood Dale, USA). Parameters were time spent in inner field (% of time), number of entries into inner field, distance traveled (m), number of bouts and time spent grooming (s), number of bouts and time spent rearing (s), and average speed (cm/s).

An animal was recorded to have entered either the inner- or outer field of the test area when all four paws had been placed inside either zone. The protocol stipulated that a rat would be excluded from analysis if a noise or other disruption occurred in the test environment, as a likely influencer of the rat’s behavior.

#### Elevated plus maze testing

At least 7 days after conducting OF testing on the rats, the rats’ exploratory behavior in the EPM was recorded using the same recording equipment described above. The EPM was constructed from black and clear Perspex and consisted of two open arms (110 cm long × 11 cm wide) and two closed arms (110 cm long × 10 cm wide × 40 cm high), extending from a central platform, which was raised 50 cm from the ground. The testing room was illuminated evenly with white light (intensity 70 lx). The protocol was run as previously described (Walf and Frye 2007). Briefly, rats were placed facing the same open arm in the central platform and exploratory behavior was recorded for 5 min. Animals were excluded from analysis under the following conditions: when a rat fell over the edge of the open arms of the maze and if a noise or other disruption caused the rat to freeze during testing. The parameters analyzed were time spent in open arms (% of time), number of entries into open arms (*n*), distance traveled (*m*), number (*n*) and time spent grooming (s), number (*n*) and time spent rearing (s), and average speed (cm/s). ANY-maze software was used to analyze the videos.

The EPM testing protocol stated that entry into an arm was only scored when all four paws of the animal had entered an arm, while a rat that fell over the edge of the open arms of the maze or froze during testing due to a noise or other disruption would be excluded.

#### Light/dark box testing

Rodents prefer dark and enclosed places, as a strategy for reducing the risk of predation (Crawley and Goodwin 1980). With this regard, the LDB assay has been deemed an ethologically relevant measure of anxiety-like behavior in rodents as it assesses approach versus avoidance behavior, baseline vigilance and defensive behaviors (Lezak et al. 2017). Both groups of rats were subjected to an LDB test paradigm at least 7 days after completing testing in the EPM. We utilized an LDB apparatus similar to that described by Bourin and Hascoe (2003), consisting of a plastic box divided into two equally sized partitions: a light—(brightly lit with white walls) and dark (minimally lit with black walls) chamber. The chambers were divided by means of a Perspex partition containing an opening that was large enough for an adult male rat to transverse between the two chambers, with the opening that was covered by a sliding door. All ChAT::Cre+ and ChAT::Cre− rats were tested in the same testing box. Testing was performed in accordance with previously described procedures (Arrant et al. 2013). The test commenced with a rat placed in the dark half of the box, with the sliding door that was raised simultaneously. Each animal’s behavior was recorded for 5 min using the recording equipment described above. Through the use of ANY-maze software, we analyzed the recordings for the parameters: (1) the latency to enter the light chamber, (2) total time spent in the light chamber and (3) the number of entries into the light chamber.

An entry into either the light or dark chamber was scored when all four paws of the animal had been placed into the respective chamber to be considered an *entry*. The only exclusion criteria that had been preset were conditions of unforeseen loud noise or other disturbances, for which the protocol stated that a subject would then be removed from the analysis.

#### Five-choice serial reaction time task

The 5-CSRTT is a well-validated task for assessment of several cognitive functions and manifesting behaviors including vigilance, impulsivity and compulsivity (Bari et al. 2008). Training and testing took place in Bussey-Saksida Touch System chambers (Lafayette Instrument, Indiana, USA) networked to a computer using ‘Whisker’ software. Chambers were trapezoidal [30 cm (height) × 35 cm (length) × 30 cm front end (width) × 25 cm rear end (width)], with a touch-sensitive liquid crystal display flat screen placed at the front end. A food magazine was placed at the rear end, into which 45 mg reward sugar pellets (Sandown Scientific, Middlesex, UK) were dispensed from an external dispenser. Black perspex masks with five evenly spaced square apertures located basally in the rat’s immediate field of vision were secured in front of the touch screens. Infrared sensors were located at the front and rear of the chamber.

Briefly, animals were trained to nose-poke into one of five briefly illuminated apertures, spaced randomly. The baseline and training task ended after 30 min or when animals had completed 100 trials. A stimulus duration (SD) of 0.5 s is standard for 5-CSRTT when assessing rats. However, behavioral output was largely below criteria (≥ 70% accuracy and ≤ 20% omissions) at an average SD of 0.5 s, so an SD of 0.6 s was instead utilized as the baseline level. Four ChAT::Cre+ and two ChAT::Cre− animals were excluded from further testing due to failure to reach final criteria. After establishing that baseline criteria were consistently met, rats were assessed using a varied SD probe (0.15, 0.3 and 0.6 s). Variables recorded at each session included: (1) correct responses; (2) incorrect responses; (3) accuracy (number of correct responses divided by the total of correct and incorrect responses, expressed as a percentage); (4) omitted responses; (5) premature responses [responses made during intertrial interval (ITI)]; (6) perseverative responses (repeat responses made after a correct response); (7) correct response latency (time from stimulus presentation until a correct response was made); (8) incorrect response latency (time from stimulus presentation until an incorrect response was made); and (9) magazine latency (time to collect reward post-correct response).

Although environmental noise was kept to a minimum, the 5-CSRTT protocol stated that in case of an unexpected noise or other disturbance occurring during 5-CSRTT testing, a rat affected would be removed from analysis.

### Animal killing and tissue handling for molecular analyses

At the end of the behavioral assessments, the rats were killed humanely by exposure to CO_2_ and the heads were decapitated. The brains were rapidly removed from the skull, immediately followed by bilateral microdissection of the prefrontal cortex (PFC) brain tissue on a prechilled metal plate. All dissection instruments were prechilled in powdered dry ice. The specimens were then flash frozen in liquid nitrogen and stored at − 80 °C until further use within labeled tubes that were immediately sealed. All steps in the procedure were performed rapidly to avoid RNA and protein degradation.

#### Contextual fear conditioning task

The CFCT is a well-validated behavioral assessment measure that is used for exploring the neural substrates involved in emotional learning and memory (Tavote et al. 2015; Kim and Jung 2006). Here, we utilized this Pavlovian conditioning task to monitor HPA-axis functioning in ChAT::Cre+ compared to ChAT::Cre− rats, by measuring levels of blood plasma markers to an acute stressor. Acquisition and retention testing took place in operant conditioning chambers (Med Associates, Vermont, US) measuring 30.5 cm (length) × 32.5 cm (height) × 24.1 cm (wide), with a plexiglass door and ceiling and metal paneling on the back and on the sides. The floor of the chamber consisted of a metal grid through which a mild electrical shock (115 VAC, 60 Hz; ENV-224AMWN, Med Associates) was delivered. Testing chambers were placed in sound and light attenuating boxes that had been networked to a computer using ‘Whisker’ software (Campden Instruments Ltd., Loughborough, UK) (Cardinal and Aitken 2010). On the day of training, each rat was placed in the chamber for 3 min, during which time it received no stimulation or cues. At the end of the third minute, rats received 3× 1 s foot shocks of 1 mA intensity with 60 s intershock intervals. Animals were removed 30 s after the final foot shock, which placed acquisition duration at 330 s. Retention testing took place 24 h later, with each animal being placed in their respective chamber and their behavior recorded for 8 min. Freezing behavior (lack of movement, except for breathing) was analyzed using Matlab software (MathWorks, Massachusetts, US). Blood plasma samples were collected from each rat to represent the baseline, acquisition and retention phases of the CFCT, with procedural details provided below.

### Blood plasma analysis of corticosterone, leptin and glucose levels

Blood was taken sublingually under general anesthesia (5% isoflurane). Baseline samples were collected 2 days prior to initiating the task protocol, at the same time of day that blood would be collected on contextual fear conditioning testing days, which occurred 25 min post-acquisition training and also the same length of time post-retention testing. Approximately, 1 ml samples were taken using ethylenediaminetetraacetic acid (EDTA)-primed vials (Thermo Fisher Scientific, UK), which were then placed on ice and centrifuged at 3000×*g* at 4 °C for 20 min. Plasma was aliquoted into Eppendorf tubes for corticosterone level analysis. Corticosterone (Cat No. ADI-901-097, Enzo Life Sciences, Devon, UK; RRID: AB_2307314) and leptin (Cat No. ADI-900-015A, Enzo Life Sciences, UK) quantification was done using enzyme-linked immunoassay (ELISA) kits with glucose quantification that was done using a colorimetric assay kit (Cat No. 10009582, Cayman Chemical, Michigan, USA), all according to the manufacturer’s instructions. The plasma corticosterone and leptin concentration levels are given as ng/ml, while glucose plasma concentration is given as mg/ml.

### Quantitative RT-PCR

To examine the copy number of mouse *VAChT* transgene, we used primers specific for the mouse *VAChT* gene (VAChT-intron forward: GAGAGTACTTTGCCTGGGAGGA and VAChT-intron reverse: GGCCACAGTAAGACCTCCCTTG) and actin primers (actin forward: TATCCTGGCCTCACTGTCCA and actin reverse: AAGGGTGTAAAACGCAGCTCA), for normalization. Actin primers were able to efficiently amplify both mouse and rat genes. Both primer pairs showed efficiency above 95% and similar amplified fragment size. We compared amplification of Cre+ rat DNA to Cre− rat DNA and also mouse DNA. Briefly, DNA was isolated from either rat (ChAT::Cre+*, n* = 5; ChAT::Cre−, *n* = 8) or mouse (*n* = 4) PFC brain tissue samples that had been pooled according to the genotype, using the phenol–chloroform extraction protocol (Guzman et al. 2011), precipitated with isopropanol and washed twice on 70% EtOH. The DNA pellet was dissolved in ultra-pure water and concentration was determined based on Nanodrop measurements. PCR reaction was prepared using the Sensifast SYBR Green kit (Cat No. BIO-98020, Bioline, UK). We used a 10-μl total volume, with each primer at concentration of 200 nM and a genomic DNA concentration of 10 ng. Technical triplicates were applied and melting curve was checked for each run. Quantitative PCR (qPCR) was performed, using the C1000TM Thermal Cycler, CFX96 Real-Time System (Bio-Rad) at the following settings: step 1: 95 °C for 5 min; step 2: 95 °C for 30 s; step 3: 60 °C for 10 s; step 4: 72 °C for 10 s; step 5: plate reading. Steps 2–5 were repeated 40 times. Copy number of mouse *VAChT* transgene in rats was determined by the 2^−ΔΔCq^ method (Livak and Schmittgen 2001). The mean *C*_q_ of the mouse *VAChT* gene and the mean *C*_q_ of actin gene in the ChAT::Cre+ and ChAT::Cre− rats were calculated with fold-change that was determined using the 2^−ΔΔCq^ method. One group was used for testing in the 5-CSRTT (ChAT::Cre+, *n* = 13; ChAT::Cre−, *n* = 8), whilst the second group was subjected to anxiety-profiling tasks, consisting of the OF arena, EPM and light/dark box (LDB) (ChAT::Cre+, *n* = 8; ChAT::Cre−, *n* = 10).

For quantifying *VAChT* mRNA expression, total RNA was isolated using the Aurum Total RNA Fatty and Fibrous Tissue Kit (Cat no. 7326830, Bio-Rad, Hercules, CA, USA), in accordance with the manufacturer’s instructions, using the remainder of the genotype-pooled rat PFC samples (ChAT::Cre+, *n* = 5; ChAT::Cre−, *n* = 7). cDNA was synthesized using a High Capacity cDNA reverse transcription kit (Thermo Fisher Scientific, UK), again following the manufacturer’s manual. cDNA was then diluted and the quantitative Real-Time PCR (RT-PCR) reaction was performed on a C1000™ Thermal Cycler, CFX96 Real-Time System (Bio-Rad) using Bioline Sensifast SYBR Green (BIO-98020) using the following PCR cycling conditions: step 1: 95 °C for 5 min; step 2: 95 °C for 10 s; step 3: 60 °C for 10 s; step 4: 72 °C for 10 s; step 5: plate reading, with steps 2–5 repeated 40 times. The primer pairs were as follows: mouse VAChT-exon forward: CCCTTTTGATGGCTGTGA, mouse VAChT-exon reverse: GGGCTAGGGTACTCATTAGA. Rat β-actin was used as reference gene for data normalization, as the actin primer can detect both rat and mice mRNA and genomic DNA. For detecting actin, the following primer pairs were used: rat β-actin forward: TATCCTGGCCTCACTGTCCA, rat actin reverse: AAGGGTGTAAAACGCAGCTCA. Technical triplicates were applied and melting curve was checked, as a standard procedure for each plate.

### Western immunoblotting

The PFC rat brain tissue samples, pooled according to genotype, were lysed using radioimmunoprecipitation assay (RIPA) lysis buffer, followed by separation of proteins on 10% SDS-PAGE (sodium dodecyl sulfate-polyacrylamide) gels. Proteins were transferred to Immobilon-P polyvinylidene difluoride (PVDF) membranes (Cat No. IPVH00010, Millipore, UK) using a semi-dry transfer apparatus (Trans-Blot Turbo™, Bio-Rad) run at 20 V for 1 h. The membrane was then blocked in 5% (w/v) powdered skimmed milk in Tris-buffered saline/0.1% Tween 20 (TBS-T) for 1 h, followed by incubation overnight at 4 °C with a primary antibody raised against α-VAChT (1:1000, rabbit polyclonal, Cat No. 139-103, Synaptic Systems, Göttingen, Germany; RRID: AB_10893979) diluted in blocking solution. The next morning, after washing 3× in TBS-T, the membrane was incubated with the secondary antibody for 1 h at RT. The membrane was then washed again in TBS-T before visualizing the signal using the ECL-plus kit (Cat No. RPN2236, GE Healthcare, UK) in the Bio-Rad Chemi-Doc^Tm^ Imaging system. Following such washes, the membrane was stripped and then probed for α-synaptophysin using a rabbit polyclonal antibody (1:2000, Cat No. 32594, Abcam, UK; RRID: AB_778204), dissolved in 5% bovine serum albumin (BSA)/TBS-T, as an internal loading control. Protein densities on Western blots were analyzed and quantified with v. 1.45 s ImageJ software (http://imagej.nih.gov/ij/; provided in the public domain by the National Institutes of Health, Bethesda, MD, USA).

### Statistical analysis

All data are expressed as mean ± standard error of the mean (SEM). SPSS Statistics (v.22, IBM) software was used for statistical analysis of the data. The distribution of the data was examined using the D’Agostino–Pearson omnibus test. Mouse *VAChT* gene copies and VAChT protein comparisons between genotypes were analyzed using an independent Student’s *t* test. A one-way ANOVA followed by the Tukey’s multiple comparison test was used to analyze *VAChT* mRNA levels between ChAT::Cre+ rats, ChAT::Cre− (Wt) rats, and also Wt mice, which served as an additional control for the VAChT mRNA measures. Corticosterone, leptin and glucose data were analyzed a using mixed-effects ANOVA with time-point being the within-subjects factor and genotype the between-subjects factor. Freezing scores for fear conditioning and all anxiety profile task parameters were compared using an independent samples *t* test. Baseline 5-CSRTT data were analyzed in a mixed-effects ANOVA with session as within-subjects factor and genotype as between-subjects factor. The varied SD probe was analyzed similarly with session and SD time as within subject and genotype as between subject factors. Any main effects were examined using the Tukey’s HSD (honestly significant difference) post hoc comparison, with interactions that were explored using a Fisher’s LSD (least significant difference) post hoc analysis; from both tools, the effect size (*η*^2^) was calculated. Any extreme outliers were identified using the ‘outlier labeling rule’ and removed from data analysis (Hoaglin et al. 1986). The rule entails that the difference between the lower and upper quartile is multiplied by a factor ‘*g*’. Due to the published advice advising that the initially suggested ‘*g*’ value of 1.5 (Hoaglin et al. 1986) proved inaccurate in some instances and that a multiplier value of 2.2 generates valid results more often (Hoaglin and Iglewicz 1987), we utilized a ‘*g*’ value of 2.2 to the datasets generated for the current project. *p* values were designated as: ***p < 0.001, extremely significant; **p ≤ 0.01, highly significant; *p ≤ 0.05, significant and *p* > 0.05, non-significant (NS).

## Results

### ChAT::Cre+ rats exhibit an anxiolytic-like and hypermobile behavioral profile

**Fig. 1:**
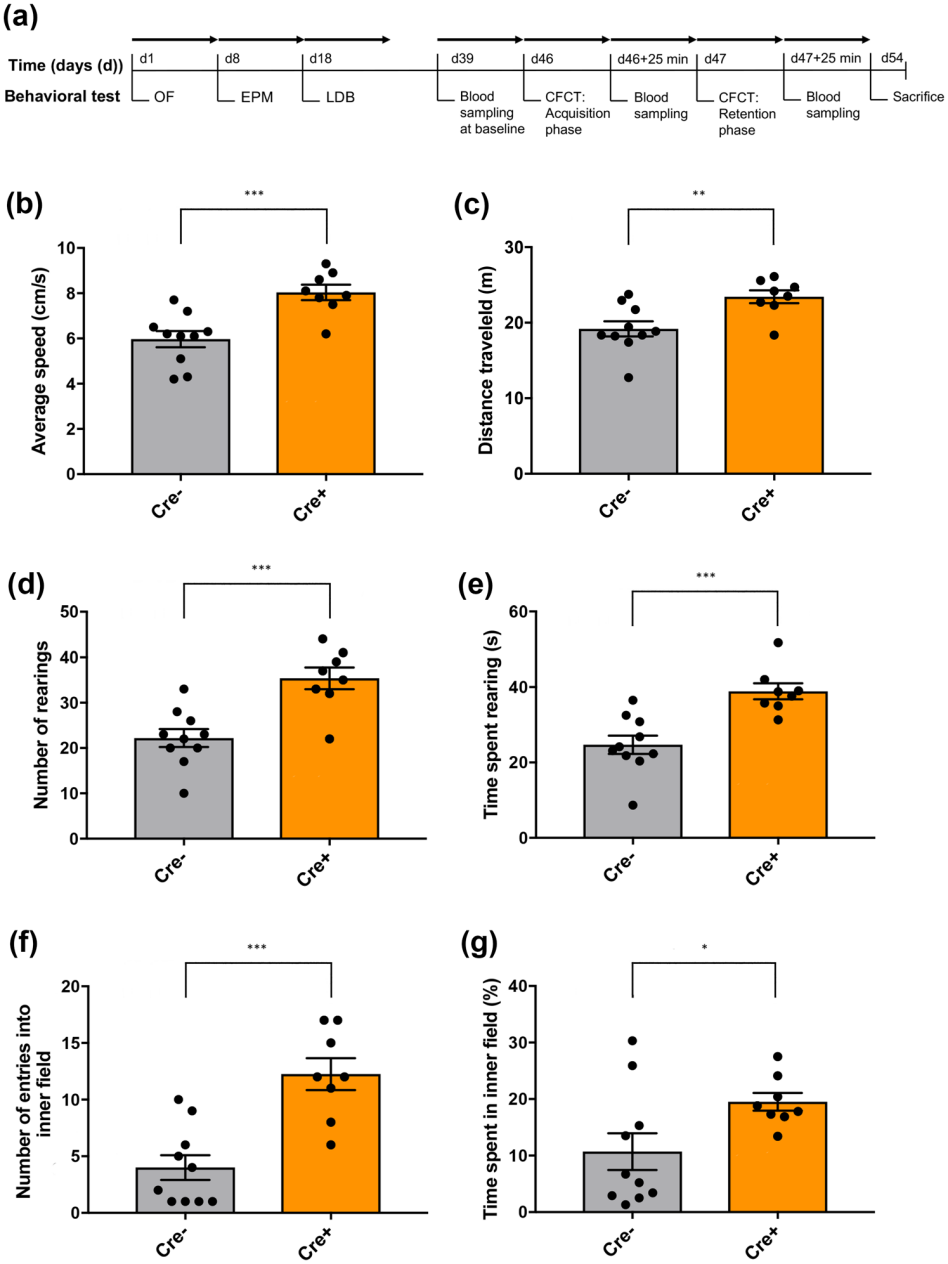
The rats’ behavior was assessed for 10 min in the OF arena to measure locomotion and anxiety-like behavior. **a** The timeline of the anxiety-related and locomotion test procedures performed on the rats are shown; all rats performed the same behavioral tests in the order shown. To investigate the acute effect of foot shock at post-acquisition and post-retention stage on plasma corticosterone, leptin and glucose levels, blood samples were collected at the time-points shown, to compare to baseline levels. In the OF test, the behavioral profile of ChAT::Cre+ rats (*n* = 8) differed from their ChAT::Cre− siblings (*n* = 10). Taken together, the parameters **b** average speed of movement, **c** total distance traveled, **d** number of rearings made and also **e** the time spent rearing during the test, indicated that ChAT::Cre+ rats were hypermobile. Other parameters like **f** number of entries into inner field and (g) time spent in the inner field, further revealed that ChAT::Cre+ rats were in a mixed hypermobile and anxiolytic state. All data are presented as the mean ± SEM. *p ≤ 0.05, **p ≤ 0.01 and ***p < 0.001, compared to ChAT::Cre− rats

Anxiety profiles were evaluated from assessing the rats’ behavior in the OF, EPM and the LDB. The behavioral time-line for anxiety profiling is shown in Fig. 1a. In the OF, ChAT::Cre+ rats displayed a hypermobility profile compared to ChAT::Cre− siblings, with a significantly increased average traveling speed (***p = 0.0008) (Fig. 1b), a greater distance covered (**p = 0.0063) (Fig. 1c), as well as rearing more often (***p = 0.0006) (Fig. 1d) and, relatedly, spending more time rearing (***p = 0.0006) (Fig. 1e). The ChAT::Cre+ also appeared anxiolytic by entering the inner diameter significantly more than ChAT::Cre− siblings (***p = 0.0002) (Fig. 1f), while also spending significantly more time within the inner field of the test arena, compared to the ChAT::Cre− rats (***p = 0.0002) (Fig. 1g).

**Fig. 2:**
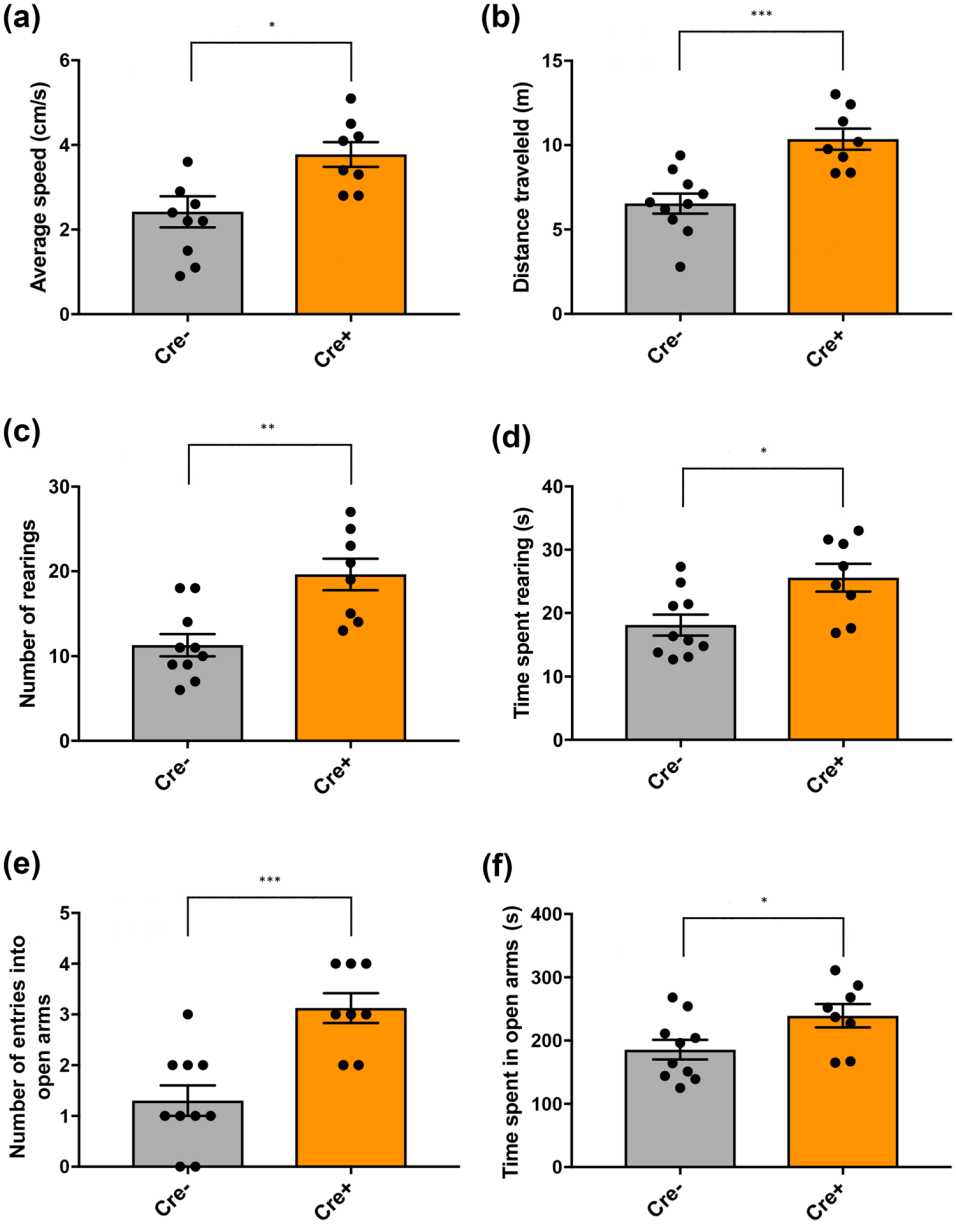
Anxiety-like behavior in the rats was further assessed in the EPM maze, where the task is based on an approach-avoidance conflict and greater avoidance of the open arms is interpreted as an exhibition of higher anxiety levels. The findings reflected those obtained for OF testing, with regards to both general ambulation and anxiety-like behavior. Over the duration of the test (5 min), ChAT::Cre+ rats (*n* = 8) traveled on average at **a** greater speed and **b** covered a greater distance than the ChAT::Cre− rats (*n* = 10). Compared to ChAT::Cre− rats, ChAT::Cre+ ones also revealed hypermobility by making **c** significantly more rearing bouts and **d** spending more time rearing. ChAT::Cre+ rats also made **e** more entries into the open arms of the EPM apparatus and **f** spent more time in the open arms than their ChAT::Cre− siblings, reflecting the general anxiolytic profile detected for the ChAT::Cre+ rats seen during testing in the OF. All data are presented as the mean ± SEM. *p ≤ 0.05, **p ≤ 0.01 and ***p < 0.001, compared to ChAT::Cre− rats

Similarly, ChAT::Cre+ rats showed higher average speed of movement (*p = 0.014) (Fig. 2a) and covered more distance in the EPM than their ChAT::Cre− siblings (***p = 0.0005) (Fig. 2b) over the entire 5 min that rats were tested in the EPM; these findings again suggested a general hyperlocomotion profile. In this test, a similar trend was also seen to that in the OF test, for ChAT::Cre+ rats to rear more (**p = 0.0017) (Fig. 2c) and for longer (*p = 0.0134) (Fig. 2d) for the full testing period within the EPM, than was seen for ChAT::Cre− rats. ChAT::Cre+ rats’ general anxiolytic tendency was reinforced by displaying an increased tendency to visit the open arms of the EPM more often than ChAT::Cre− rats (***p = 0.0006) (Fig. 2e) and spending significantly more time in the open arms of the maze (*p = 0.0403) (Fig. 2f) compared to ChAT::Cre− rats.

**Fig. 3:**
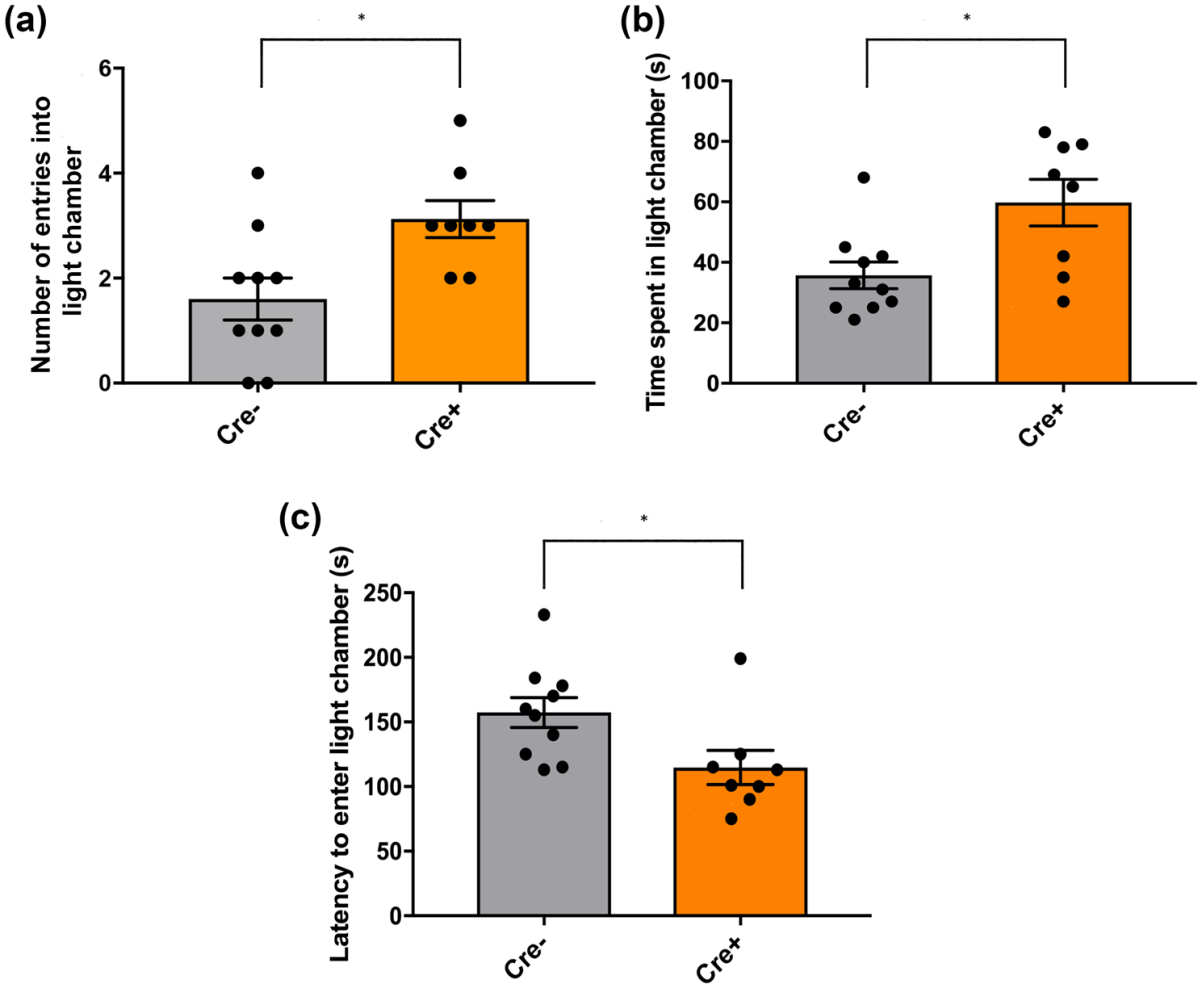
The LDB is another conventional anxiety test that is based on the innate aversion of rodents to brightly illuminated areas, which was utilized to test unconditional anxiety responses in the rats. The ChAT::Cre+ rats (*n* = 8) showed a greater preference for the light compartment of the box compared to their ChAT::Cre− siblings (*n* = 10). Behavioral parameters assessed were: **a** total number of entries made into the light chamber, **b** time spent in the light chamber and **c** latency time for rats to enter the light chamber. All data are presented as the mean ± SEM. *p ≤ 0.05, compared to ChAT::Cre− rats

In the LDB, ChAT::Cre+ rats entered the light chamber significantly more frequently (*p = 0.0132) (Fig. 3a), exhibited greater average duration of time in the light chamber (*p = 0.0118) (Fig. 3b) and subsequently, also spent less time in the dark chamber of the test apparatus (*p = 0.0277) (Fig. 3c). Taken together, use of multiple experimental paradigms revealed that the ChAT::Cre+ rat model displays spontaneous locomotion and adaptation to a novel, anxiety-evoking environment that differs from the baseline behavioral profile seen for non-transgenic littermate rats of a similar strain.

None of the rats’ performance scores were excluded from the final analysis for the anxiety-profiling tasks, based on the exclusion criteria set for each behavioral measure (for a description of these, please see the “Materials and methods”). In addition, no animal died during behavioral testing. Hence, data collected for all rats tested in the OF, EPM and LDB were included in the final analyses.

ChAT::Cre+ rats make more errors of omission and fewer perseverative responses than Chat::Cre− rats in the 5-CSRTT.

**Fig. 4:**
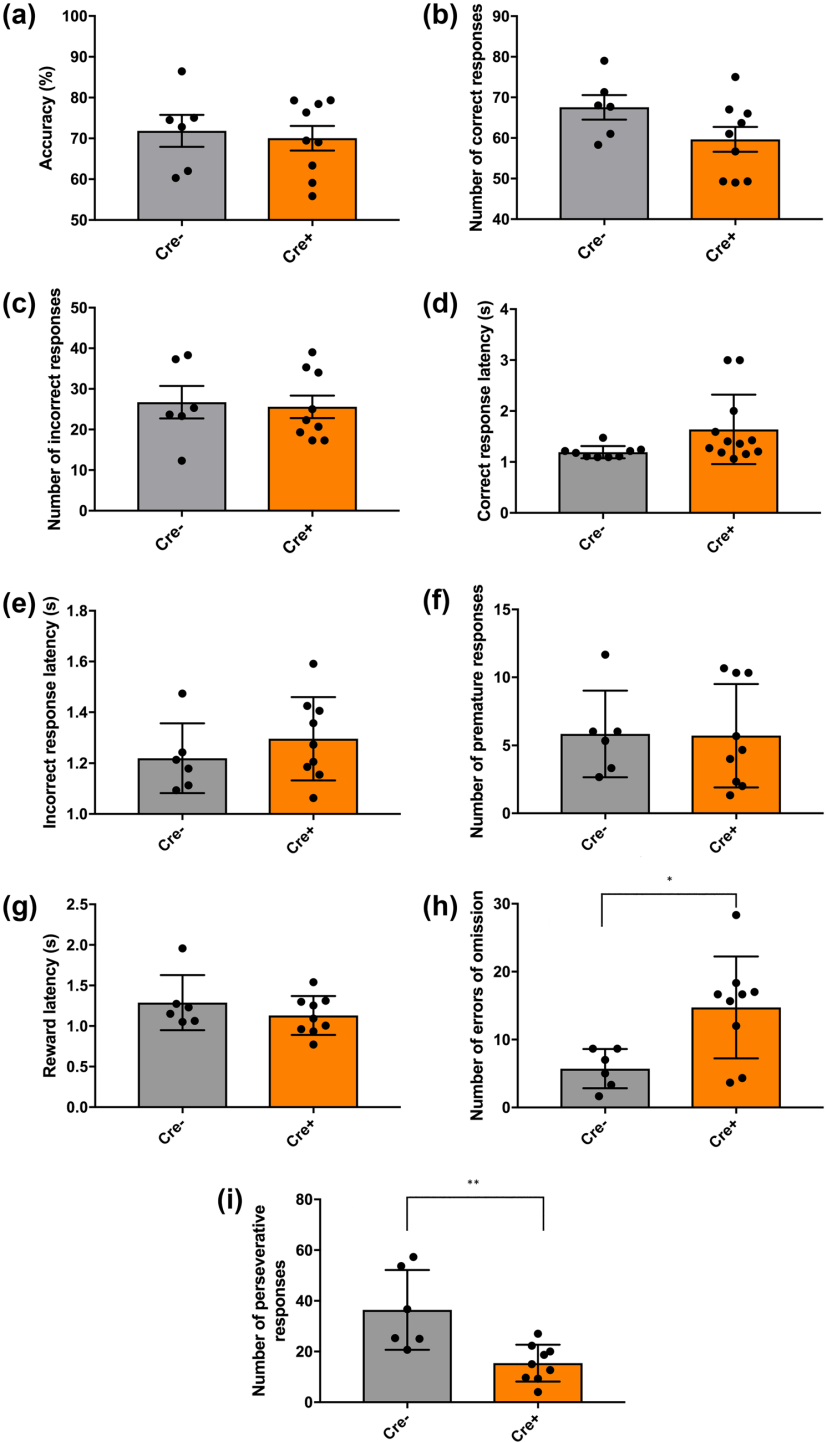
Performance in the 5-CSRTT assessed for differences in attention, impulsivity and cognitive flexibility between the transgenic and Wt rats. ChAT::Cre+ rats (*n* = 13) made significantly more omission errors and fewer perseverative responses than their ChAT::Cre− siblings (*n* = 8) during 5-CSRTT testing, which measured the following parameters: **a** choice accuracy; **b** average number of correct responses; **c** average number of incorrect responses; **d** latency to make a correct nose poke response following stimulus presentation; **e** latency to make an incorrect response following stimulus presentation; **f** average number of premature responses; **g** latency to collect sugar pellet reward from food magazine after a correct response; **h** average number of errors of omission and **i** average total number of perseverative responses. All data are presented as the mean ± SEM. *p ≤ 0.05, **p ≤ 0.01, compared to ChAT::Cre− rats

Figure 4 summarizes the results of the behavior displayed by the two genotypes in the 5-CSRTT. No differences were detected between the two groups in terms of choice accuracy (*p* = 0.714, NS) (Fig. 4a), the number of correct responses (*p* = 0.105, NS) (Fig. 4b) or the number of incorrect responses (*p* = 0.814, NS) (Fig. 4c). Relatedly, the measures ‘mean delay to make a correct response’ (*p* = 0.364, NS) (Fig. 4d) or to make an incorrect response (*p* = 0.061, NS) (Fig. 4e) were also left unchanged between transgenic and non-transgenic rats. Furthermore, the measures ‘number of premature responses’ (*p* = 0.946, NS) (Fig. 4f) and ‘mean latency of reward collection following a correct response’ (*p* = 0.308, NS) (Fig. 4g) were also similar between ChAT::Cre+ and ChAT::Cre− rats. However, compared to their ChAT::Cre− siblings, ChAT::Cre+ rats made significantly more errors of omission (*p = 0.016, *η*^2^ = 0.373) (Fig. 4h), but fewer perseverative responses (**p = 0.006, *η*^2^ = 0.456) (Fig. 4i).

**Fig. 5:**
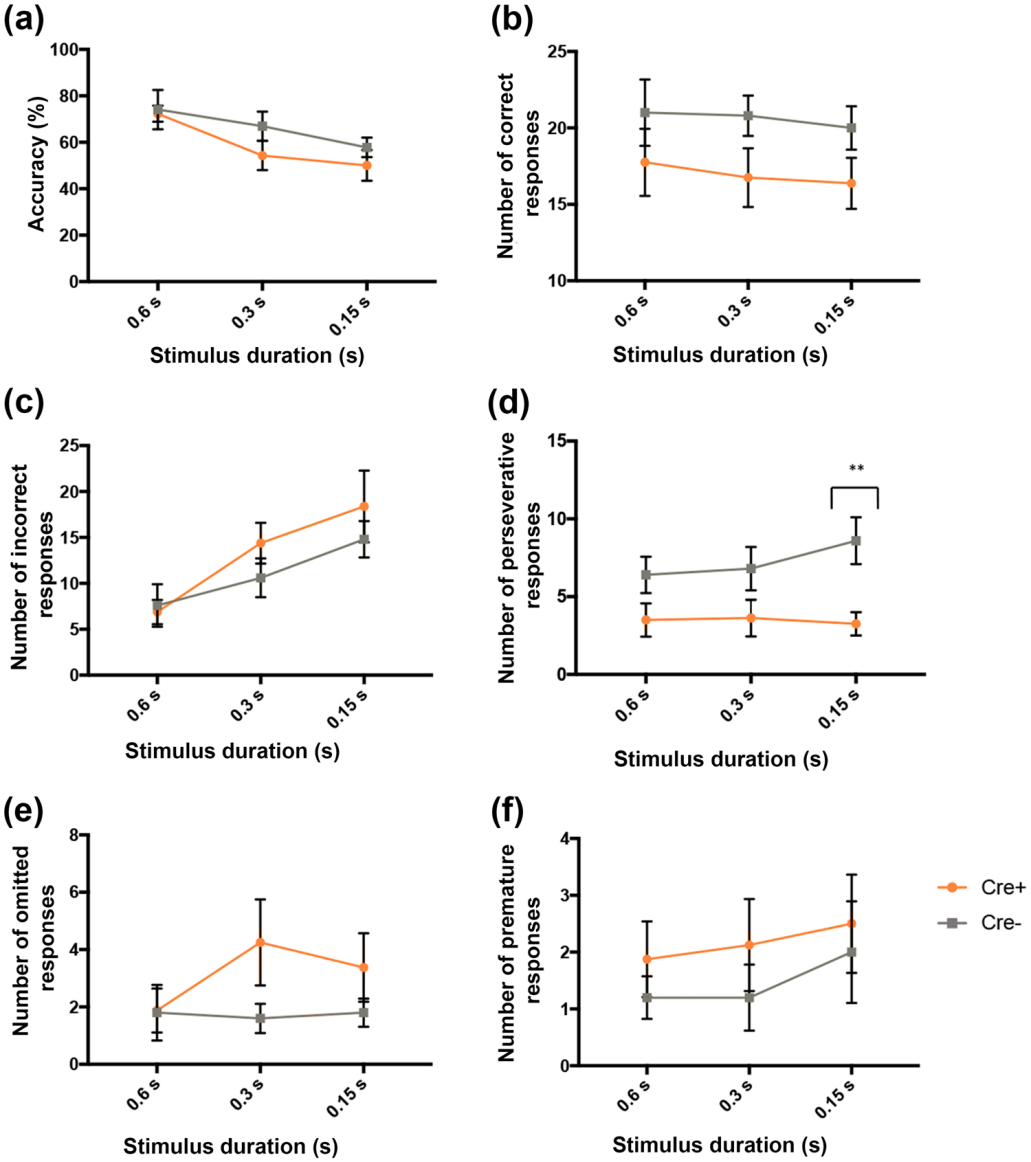
A version of the 5-CSRTT, where SD length was varied (0.6, 0.3 and 0.15 s), further measured attentiveness, impulsivity, processing speed and distractibility in the rats. SD length variance exerted an overall significant effect on **a** choice accuracy. This was since, compared to ChAT::Cre− rats (*n* = 8), the ChAT::Cre+ ones (*n* = 13) made on average, **b** less correct responses and, therefore, **c** more incorrect responses, as SD decreased. **d** ChAT::Cre+ rats made less perseverative responses compared to ChAT::Cre− ones, with the number of perseverative responses that decreased most significantly at the longest duration probe, namely 0.15 s. **e** As detected in the standard 5-CSRTT, a tendency by ChAT::Cre+ rats to omit more responses compared to baseline behavior was not repeated. **f** Groups revealed similar levels of premature responses. All data are presented as the mean ± SEM. **p ≤ 0.01, compared to ChAT::Cre− rats

During a version of the 5-CSRTT, where the SD probe length was varied, a main effect of SD on accuracy was detected (**p = 0.002) (Fig. 5a) that featured as decreasing accuracy as SD times became shorter. This effect was caused by the overall tendency of ChAT::Cre+ rats to make fewer correct responses (*p = 0.05) (Fig. 5b), implying more incorrect responses (**p = 0.009) (Fig. 5c) as the SD decreased, compared to ChAT::Cre− siblings. However, no significant interaction was detected between SD variation and genotype. Between-subject genotype analysis showed that upon variable and, therefore, increased demand conditions, ChAT::Cre+ rats made fewer perseverative responses compared to ChAT::Cre− ones (*p = 0.018, *η*^2^ = 0.410) (Fig. 5d). This was similar to baseline behavior, to indicate that this phenotypical difference could be regarded as robust. When comparing perseverative responses assessed for different SD times, this decrease was significant at the longest duration probe tested for, namely 0.15 s (**p = 0.005) (Fig. 5d). The tendency for ChAT::Cre+ rats to omit more responses compared to their baseline behavior was not seen in the varied SD probe (*p* = 0.287, NS) (Fig. 5e). Differences were also not seen between genotypes in the varied SD probe when number of premature responses were assessed (*p* = 0.389, NS) (Fig. 5f).

### ChAT::Cre+ rats display a biologically intact stress response to an acute stressor in a CFCT paradigm

Previous studies showed that the cholinergic system could play a role in HPA activation and corticosterone levels (Newman et al. 2001; Paul et al. 2015). To investigate the effects of altered cholinergic modulation on physiological stress responses, we measured plasma levels of corticosterone at baseline and post-retention testing in the CFCT. With this regard, several studies have demonstrated a positive relationship between circulating corticosterone levels and contextual conditioning (Pugh et al. 1997; Thompson et al. 2004).

**Fig. 6:**
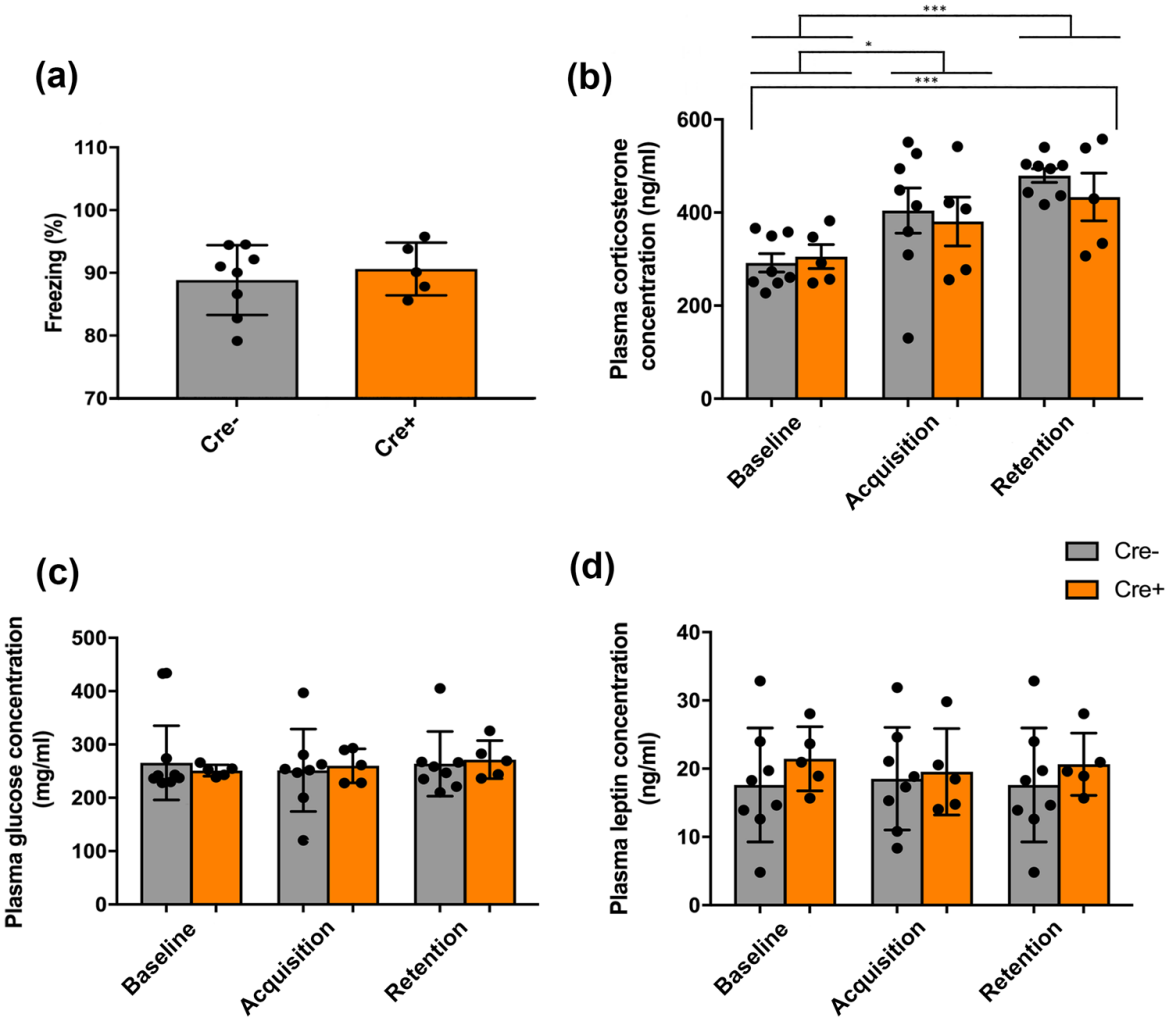
Freezing behavior and blood plasma analysis of HPA-axis biomarkers performed on ChAT::Cre+ (*n* = 5) and ChAT::Cre− (*n* = 8) rats at baseline (2 days before initiating the task protocol), with testing that was then repeated at the fear acquisition and fear retention stages of a CFCT. **a** Freezing scores acquired for retention testing; **b** comparison of corticosterone blood plasma levels between genotypes at the different CFCT testing phases; **c** a similar comparison made for glucose blood plasma levels and for **d** leptin blood plasma levels. The significant increase in blood plasma corticosterone levels seen in both rat genotypes from baseline to a repeat analysis soon after rats completed the fear acquisition and also the fear retention test phases, indicates that all rats of this strain, regardless of the genotype (ChAT::Cre+ vs. Wt), respond in a biologically intact manner to aversive conditioning. All data are presented as the mean ± SEM. *p ≤ 0.05 and ***p < 0.001, compared to both genotype rats’ baseline measurements

The level of freezing, measured during retention testing, did not show any significant difference between genotypes (*p* = 0.642, NS) and produced an average of ~ 90% for each group (Fig. 6a). When all rats were considered together, without accounting for genotype, a significant increase of corticosterone from baseline to fear acquisition was seen (*p < 0.05), and also when baseline levels were compared to levels reached at the retention phase of the CFCT (***p < 0.001) (Fig. 6b). However, this effect was independent of genotype (*p* = 0.618, NS), with no interaction seen between genotype × sample point either (*p* = 0.199, NS). Post hoc analysis revealed high statistical significance between differential levels of circulating corticosterone levels from baseline to retention phase for ChAT::Cre− litter mates (***p < 0.001); however, a similar comparison made for ChAT::Cre+ rats proved non-significant (*p* = 0.057, NS) (Fig. 6b).

Analysis of plasma glucose levels revealed no significant difference between sample points (*p* = 0.196, NS), when comparing the genotypes (*p* = 0.095, NS) or at a sample point × genotype interaction level (*p* = 0.290, NS) (Fig. 6c). Analysis of leptin levels revealed no significant difference in main effect of sample point (*p* = 0.50, NS) or of genotype (*p* = 0.933, NS), while no significant interaction effect was observed either (*p* = 0.108, NS) (Fig. 6d).

### Higher levels of VAChT mRNA and protein expression in ChAT::Cre+ rats compared to Wt rats

**Fig. 7:**
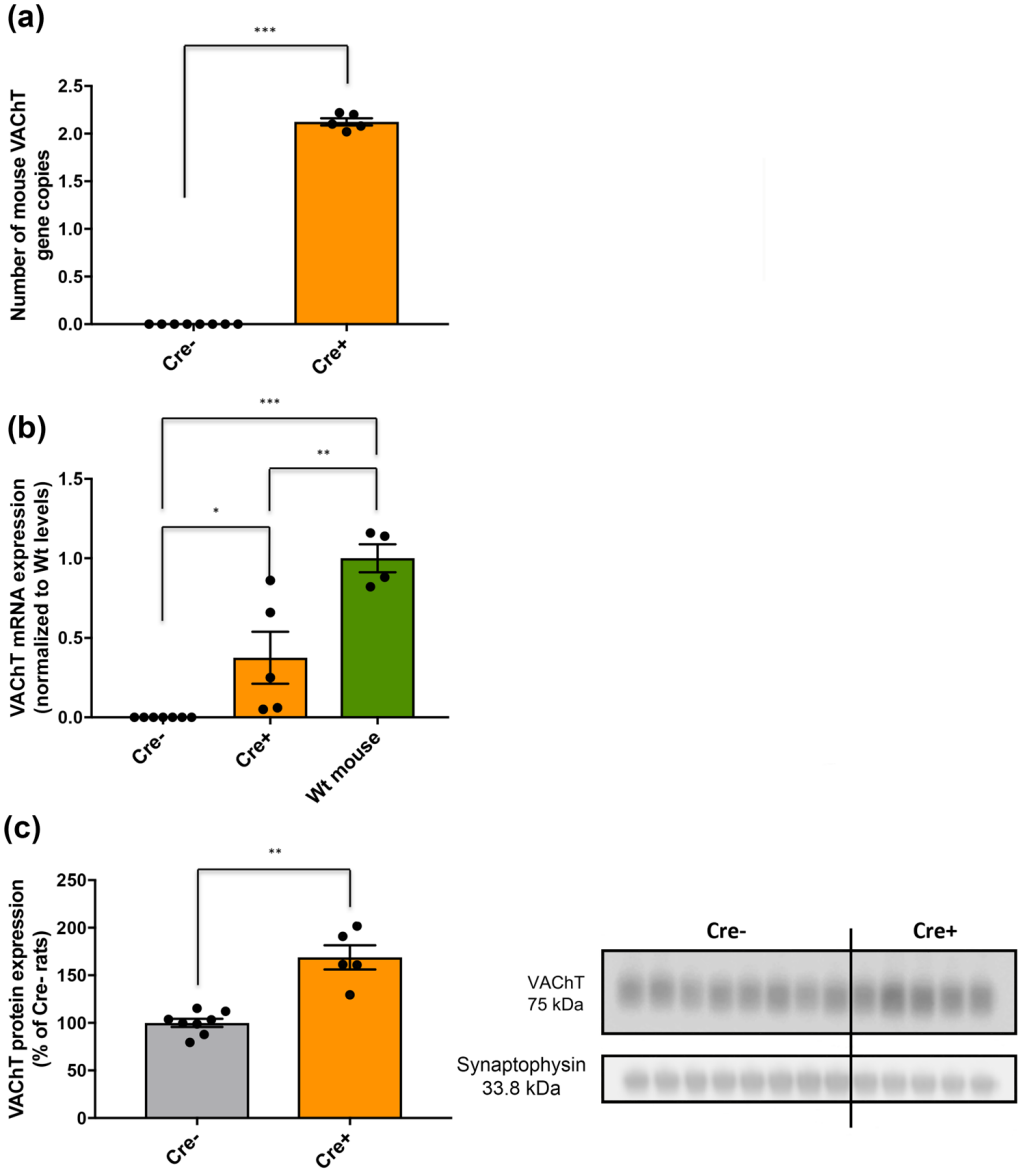
Measurement of prefrontal cortical VAChT mRNA and protein levels. **a** qPCR results indicated additional mouse *VAChT* gene copies in all ChAT::Cre+ rats (*n* = 5) compared to ChAT::Cre− controls (*n* = 8). This translated to **b** increased mouse VAChT mRNA expression in Cre+ (*n* = 5) and Cre− (*n* = 7) siblings compared to Wt mice (*n* = 4) and also **c** increased VAChT protein expression in PFC brain samples of Cre+ (*n* = 5) and Cre− (*n* = 8) rats. All data are presented as the mean ± SEM. *p ≤ 0.05, **p ≤ 0.01 and ***p < 0.001, compared to ChAT::Cre− rats and Wt mouse

qPCR results revealed that all Long-Evans ChAT::Cre+ rats analyzed here contained two copies of the mouse *VAChT* gene relative to ChAT::Cre− rats (***p < 0.001) (Fig. 7a). Mouse VAChT mRNA was variable within individual rats, with some having levels similar to mouse controls (Fig. 7b). However, overall, the expression of mouse VAChT mRNA in ChAT::Cre+ rats achieved approximately 50% the levels of mouse controls (Fig. 7b), which indicates the expression of the mouse *VAChT* gene inserts in the rat genome. Supporting the gene copy and the mRNA analysis, VAChT protein levels were increased close to 50% in ChAT::Cre+ rats when compared to Cre− siblings (**p = 0.004) (Fig. 7c). This result supports the notion that the increased *VAChT* expression found in ChAT::Cre+ rats may also increase ACh levels within synaptic vesicles, as had previously been reported for BAC transgenic mice that express channelrhodopsin protein, a light-gated ion channel, under control of the ChaT promoter (Sugita et al. 2016).

## Discussion

The current findings show that, compared to their Wt siblings, Long-Evans ChAT::Cre+ rats exhibit altered baseline behavior, affecting gross locomotor function, anxiety-related behavior as well as some aspects of sustained attention. We also molecularly analyzed the PFCs collected from the rats, with this brain region that receives dense cholinergic innervation and also connects directly and indirectly via cholinergic projection neurons with multiple brain areas, to provide important modulation of cortical function (Bloem et al. 2014; Tingley et al. 2015). The analysis revealed that these behavioral changes were paralleled by altered expression of VAChT within the PFC, at both a gene and protein level, with VAChT being a key molecular mechanism in the transport machinery of ACh from the cytoplasm to the lumen of synaptic vesicles, to ultimately release ACh into the synaptic cleft. Physiologically, an increase in levels of this functional transporter could alter ACh levels at the synapse (Kolisnyk et al. 2013; Sugita et al. 2016; Nagy and Aubert 2012), which could alter cholinergic tone and, subsequently, behavioral phenotype in rodents. Hence, the current findings demonstrate that expression of the *ChAT*-*BAC* construct makes these rats different from their Wt siblings, similar to findings previously reported using BAC transgenic mice engineered to express a light-sensitive protein under control of the ChAT promoter (Kolisnyk et al. 2013; Chen et al. 2018).

As was reviewed by Prado et al. (2013), ACh synthesis depends on the uptake of the ACh precursor, choline by CHT1 [high-affinity choline transporter/SLC5A7 (solute carrier family 5 member 7)] that mainly expresses within cholinergic neurons, yet presents in certain non-neuronal cells also. In the cytoplasm of synapses, ACh is then synthesized by the enzyme ChAT, with ACh that is then loaded into synaptic vesicles by VAChT. Upon arrival of a nerve impulse, the vesicles fuse to the plasma membrane and release ACh that signal through nicotinic and muscarinic cholinergic receptors. ACh is rapidly degraded into acetate and choline by the enzyme AChE (acetylcholinesterase). In the current work, we have only evaluated expression levels of VAChT, in line with other studies utilizing BAC transgenic mice for expressing a light-sensitive protein under control of the ChAT promoter that also restricted molecular analysis to VAChT, as a valid indicator of cholinergic tone (Kolisnyk et al. 2013; Chen et al. 2018). The importance of functional vesicular storage and synaptic “loading” of ACh, which is modulated by VAChT, acts as a required step for ACh release. In addition, decreased VAChT expression has been shown to lead to several motor and cognitive dysfunctions (Prado et al. 2006; Martyn et al. 2012), some of which was presently behaviorally evaluated for in the ChAT::Cre+.

To ensure a comprehensive understanding of the levels at which altered cholinergic metabolism and transmission might be altered as a result of the ChAT::Cre+ genotype, future work should undertake gene and protein expression analysis of other important mediators in this process, including but not limited to ChAT, CHT-1, AChE, but also muscarinic and nicotinic receptors for signaling ACh. Such eventual comprehensive profiling will inform greatly on the design of experimental work involving this popular experimental rat tool.

Our data indicated a hypermobile state for ChAT::Cre+ rats over Wt ones in several parameters assessed in the OF, EPM and LDB. The observed locomotor arousal in the transgenic rats is consistent with the previously proposed mechanism that instantaneous ACh synaptic release correlates positively with increased activity by rodents seen in novel environments (Mizuno et al. 1991; Cohen et al. 2012), while ACh is known to fulfill a complex role in locomotor control, which includes modulating the dopaminergic system (Rice and Cragg 2004). Confirmation of the specific mechanisms for the observed hyperactive spontaneous activity awaits brain region-specific VAChT overexpressing rat models.

This result differs from those presented by Azzopardi et al. (2018) who observed no hyperlocomotive phenotype in ChAT::Cre+ rats, although enhanced motor endurance had previously been reported for the ChAT-ChR2-eYFP (enhanced Yellow Fluorescent Protein) BAC transgenic mouse line (Kolisnyk et al. 2013). It is worth noting though that the study by Azzopardi et al. (2018) made use of a single metric (distance traveled) for assessing abnormal mobility, with the group using a 45 × 45 cm square OF box. Instead, we made use of a circular test apparatus, with the shape of OF arena (circular, square or rectangular) that has been identified as an important variable that can influence OF performance by rodents (Sestakova et al. 2013). Similarly, this research group only assessed the time spent in the outer perimeter in the same apparatus as indicator of anxiety. Lastly, Azzopardi et al. (2018) utilized rats in the experimental procedures at a slightly older age, at 10–14 weeks, whereas rats used in the current study ranged between 8 and 10 weeks of age. However, Long–Evans rats are considered to have reached adulthood by 8 weeks of age (Snyder et al. 2009; Jackson et al. 2016), while the age ranges of rats used in the present work do overlap somewhat with that of the study by Azzopardi et al. (2018), allowing for reasonable confidence that the differences in experimental outcomes between these two studies is likely and at least not exclusively due to age-related differences. However, differences in the age of experimental rats have been highlighted as a potentially critical experimental variable, to especially account for changes in neurotransmitter release and receptor activation, that appear to alter substantially, especially over early stages of development (McCutcheon and Marinelli 2009). Considering the differences in testing conditions and the characteristics of the experimental rats used in the current study compared to those used by Azzopardi et al. (2018), we conclude that our ambulation and anxiety results may not be directly comparable.

Our results also reveal ChAT::Cre+ rats to be anxiolytic, as was assessed in three frequently used tests of anxiety-like behavior, with such tests that consistently demonstrate the ability to reveal anxiolytic or anxiogenic profiles of rodents using either pharmacological- or transgenic manipulations (Crawley 1985; Belzung and Griebel 2001; Prut and Belzung 2003). This result suggests that modest levels of *VAChT* overexpression at both the gene and protein level, previously shown to increase ACh release and, therefore, render animals hypercholinergic (Sugita et al. 2016; Janickova et al. 2017), associate with an anxiolytic behavioral profile in the ChAT::Cre+ rats. The findings reflect those from another hypercholinergic mouse model that also overexpresses VAChT at the gene and protein level, with such mice that showed enhanced exploration of novel environments and, therefore, anxiolytic-like behavior, when compared to C57BL/6J (B6) control mice (Nagy and Aubert 2013). In this work, use was made of the B6eGFPChAT congenic mouse model that contains four genomic copies of the cholinergic gene locus, which encompasses the promoter and coding regions of both VAChT and ChAT (Tallini et al. 2006; Nagy and Aubert 2012). As earlier work demonstrated increased VAChT gene and protein expression in congenic B6eGFPChAT mice (Nagy and Aubert 2012), this experimental model allows for evaluating whether increasing the vesicular storage and release of ACh is sufficient for eliciting changes in behavioral activity.

Relatedly, previous studies showed that exposure to novel stimuli (i.e., novel environments) associates with cholinergic activation and increased ACh release (Thiel et al. 1998; Giovannini et al. 2001), while others showed that modulation of central cholinergic systems regulate emotional processes including anxiety and stress (Podhorna and Franklin 1999, 2000; Janickova et al. 2019). However, the effects of ACh on anxiety-like behavior in rodents has proved complex, with increased ACh release that evoked both anxiolytic and anxiogenic reactions (File et al. 1998, 2000), which may relate to brain region-specific configurations of ACh receptors (File et al. 2000; Labarca et al. 2001).

The cholinergic system has been shown to play an important role in attentional mechanisms. Selective lesioning of the Ch5 cholinergic cell group of the pedunculopontine nucleus (PPN) (Mesulam et al. 1984), a rostral brainstem nucleus, resulted in a deficit shown during tests assessing for sustained attention, which manifested as a significant decrease in the number of correct responses and an increase in response latency (Cyr et al. 2015). Similarly, cholinergic-specific lesions made in rats to the Ch4 cholinergic cell group located in the nucleus basalis magnocellularis (NBMc), the rat homologue of the nucleus basalis of Meynert (NBM), by means of 192 IgG-saporin infusions, also produced attentional impairments along with decreased levels of ACh in the PFC (McGaughy et al. 2002). Moreover, attentional impairments are deemed a core feature of AD and have been shown to be caused, at least in part, by damage sustained by the NBM, which undergoes significant neuronal loss during progressive AD (Ferreira-Vieira et al. 2016).

Kolisnyk et al. (2013) showed that BAC transgenic mice generated to express the light-gated ion channel, channelrhodopsin-2, under control of the ChAT promoter, with such animals that are frequently used for dissecting out the relative contribution of cholinergic circuits in behavior, display severe deficits in several domains of cognitive function, and that this may be due to VAChT overexpression seen in such mice. In particular, transgenic mice were impaired in tasks assessing the domains spatial and working memory. Interestingly, Nagy and Aubert (2015) reported, using hypercholinergic congenic B6eGFPChAT aged mice, improved spatial memory acquisition during testing in the Morris water maze, to reveal a consensus between VAChT overexpression and improved spatial navigation during normal aging. The study further demonstrated an association between the mice’s augmented memory acquisition and enhanced dendritic ramification of adult-born neurons seen in the hippocampus, and at progressive ages. Since cholinergic signaling was previously shown to contribute to the dendritic expansion of target neurons (Höhmann et al. 1991), the study by Nagy and Aubert (2015) supports a role for cholinergic input to the hippocampus as a mechanism underlying the formation of dendritic networks to support formation of spatial memories.

Kolisnyk et al. (2013) also reported that the transgenic mice exhibited significantly more premature responses during the 5-CSRTT, a behavioral paradigm frequently used for evaluating attention and impulsivity in animal models. This result differed from our current findings, which did not detect a similar deficit for the transgenic rats when subjected to either the self-paced version of the 5-CSRTT, or when the SD was varied during testing. Interestingly, increased premature responding has been associated with depletion of another monoamine neurotransmitter, serotonin (Fletcher et al. 2013; Humpston et al. 2013); hence, our results seemingly agree that a cholinergic imbalance in the brain do not manifest as this 5-CSRTT parameter.

However, the construct ‘impulsivity’ encompasses various dimensions, namely motor impulsivity which refers to acting without thinking, cognitive impulsivity referring to rapid decision taking and non-planning impulsivity, referring to a present orientation (Patton et al. 1995). Furthermore, 5-CSRTT performance differences have been reported to exist between mice and rats, including a strategy bias with rats that showed greater reliance on a temporal strategy than mice did, resulting in more guessing if their timing was poor, and manifesting as premature responses (Fletcher et al. 2007). First, for validating whether differences exist between mice and rats in 5-CSRTT performance, the protocol should be standardized, including intertrial interval (ITI) lengths. Species differences in baseline neurotransmitter concentrations could also play a role (Fitzgerald 2009). In addition, given the complexity of mechanisms underlying 5-CSRTT parameters, including impulsivity, it is unlikely that a single brain function abnormality would underlie species differences, calling for considering interacting neurochemistry systems as the plausible basis of the observed interspecies differences in 5-CSRTT performance.

Our results also show that ChAT::Cre+ rats make significantly more errors of omission than their Wt siblings, although this effect did not persist when rats were tested with a variable attentional load as a result of shorter or longer it is. Although accuracy was unaffected between the transgenic and Wt rats, this arguably points at a deficit in sustained attention, since the ChAT::Cre+ rats showed an increase in omissions, while latency parameters were left largely unaffected. Multiple studies have suggested that cholinergic signaling, especially when mediated by α4β2 nicotinic acetylcholine receptors, underlies altered responses in this attentional parameter measured in the 5-CSRTT (Shoaib and Bizarro 2005; Semenova et al. 2007). However, again multiple interacting neuromodulatory systems might form the neurochemical substrate of this deficit, as several studies suggested that enhanced midbrain dopaminergic activity in rats influenced trial omissions, although the direction of the effect was unpredictable (Granon et al. 2000; Agnoli et al. 2013; Boekhoudt et al. 2017). It has been suggested that the monoaminergic and cholinergic systems may play separable roles in different aspects of performance controlled by the 5-CSRTT (Robbins 2002). In the case of response omissions, motivational factors have been deemed important for driving this behavioral change, and not merely attentional deficits, with dopamine that plays an established role in reward-motivation behavior mechanisms (Salamone and Correa 2012).

We also found that ChAT::Cre+ rats made significantly fewer perseverative responses in the 5-CSRTT, with this parameter that is commonly utilized as a reference for compulsive behavior as well as cognitive inflexibility, defined as the inability to adapt to goal-directed behavior in response to changing environmental demands (Gilbert and Burgess 2008; Eagle and Baunez 2010). Deficits in the ability to flexibly update behavior are observed in various neurological and psychiatric conditions including PD (Cools et al. 2001), obsessive-compulsive disorder (OCD) (Chamberlain et al. 2008), drug addiction (Everitt et al. 2007), attention deficit/hyperactive disorder (ADHD) (Rubia et al. 2009) and pathological gambling (Koehler et al. 2013). The balance of evidence suggests that the PFC and striatum, which form part of the portico–basal–thalamic circuit, co-modulate cognitive flexibility, while a multitude of studies demonstrated that ACh transmission facilitates this process (Prado et al. 2017). With this regard, several studies have shown that cortical cholinergic manipulations selectively affect aspects of this cognitive domain with PFC nicotinic receptors that appear to play an important modulatory role in this process (Allison and Shoaib 2013). For example, in old rats (Tait et al. 2013; Nikiforuk et al. 2015), rat models of schizophrenia (Alexander et al. 2013), and in rats that sustained immunolesioning of the basal forebrain cholinergic neurons (Cutuli et al. 2009), systemic administration of cholinesterase inhibitors for augmenting cholinergic transmission, improved both reversal learning (learning an association exists between one stimulus (of a pair) and reward, to then learn a reverse association where a previously unrewarded stimulus is now rewarded) and attentional set-shifting (shifting from one mental set to another) impairments. Additional experimental evidence emphasizes a role by prefrontal ACh in cognitive flexibility, in particular the critical role by PFC-mediated cholinergic signaling in the initial inhibition of a previously learned strategy. Ridley et al. (1994) transplanted neocortices of marmosets with cholinergic-rich neural tissue some weeks after inducing bilateral excitotoxic lesions to the NBM, where animals showed intact reversal learning during a visual discrimination task. In addition, others showed no impaired acquisition in an operant discrimination task, but deficits in reversal learning, in rats that had received selective cholinergic lesioning of the NBMc (Cabrera et al. 2006). However, the role of cholinergic signaling and the synergistic interaction between this neurochemical system and the PFC in mediating cognitive flexibility remains controversial, with some authors reporting that ablation of basal forebrain cholinergic neurons does not impair reversal learning (McGaughy et al. 2008; Tait and Brown 2008). Additional participation in cognitive flexibility by ACh within other brain areas such as the hippocampus, basolateral amygdala and posterior parietal cortex also participate in cognitive flexibility (Prado et al. 2017) may explain these controversies; however, the modulating role played by PFC ACh in this process is strengthened by a large number of studies showing that cholinergic depletion of different prefrontal areas results in dissociable deficits in separate forms of cognitive flexibility. Optogenetic and chemogenetic tools to modulate neuronal activity levels promise to characterize the cholinergic circuits involved this and other cognitive domains and unravel the exact role of the PFC in the process. Genetically modified rodents, including ChAT::Cre+ rats as we assessed in the current study, compliment such cell- and circuit-modifying tools, by allowing cholinergic machinery to be spatially and temporally targeted. However, as our present study demonstrates, behavioral results obtained from use of such animals should consider whether baseline behavioral changes are due to a hypercholinergic phenotype.

Cholinergic tone has also been associated with several memory- and learning-related functions. In addition, cholinergic neurons of the PFC, which we dissected out in the rats for gene and protein analysis of VAChT, have been implicated as a modulator (via cholinergic signaling) of specific aspects of memory (Croxson et al. 2011). Hence, an important direction for future research is to characterize the extent and nature of memory-related impairments in this transgenic rat line.

Although the current work did not incorporate an extensive evaluation of memory-related functions in the rats, a CFCT was included, where animals learn that certain environmental stimuli predict aversive events. This classical, Pavlovian (respondent) conditioning model provides an interface between memory and emotion (LeDoux 2000). In this testing paradigm, we measured the frequency of a defensive reflex namely freezing, shown by the rats during the retention stage of the CFCT, with freezing response considered to provide a readout of memory that is dependent on the hippocampus, amygdala and PFC (McEchron et al. 1998; Gilmartin and Helmstetter 2010; Kochli et al. 2015). Hence, as this assessment entails cognitive/explicit and non-cognitive/implicit fear memory that are supported by neural pathways that converge, the CFCT rather assesses associative memory processes by testing a memory for the association between an aversive stimulus (mild foot shock) and a salient environmental cue. Significantly enhanced freezing behavior revealed upon re-presentation of the context by one genotype rat group compared to the other would have indicated enhanced learning and memory ability. However, as our results report no difference in the frequency of freezing behavior, we conclude that this memory unit was unaffected by the genotype of the rats.

Memory as a cognitive domain can be classified according to several criteria, including function (e.g., working vs. reference memory), content (e.g. declarative/explicit vs. procedural/implicit memory), duration (e.g., immediate or short-term vs. long-term or remote memory), nature (associative vs. non-associative memory), or motivation (appetitive/reward vs. aversive memory). Hence, to allow for comprehensive profiling of mnemonic function, studies aimed at assessing differences in performance between ChAT::Cre+ versus ChAT::Cre+ rats should utilize a battery of tests that cover most, if not all fundamental types of memory processes (Savage and Ma 2014).

With evidence suggesting that the HPA-axis may be dysfunctional in children with ADHD (Hong et al. 2003), and intriguing findings indicating for a molecular interactive link between the brain’s cholinergic systems and the HPA-axis neuroendocrine system in modulating cognitive processes (Paul et al. 2015), we also determined for possible defective HPA-axis activity in ChAT::Cre+ compared to Wt siblings, in response to fear conditioning. We assessed circulating levels of HPA-axis activity markers. Specifically, the stress hormone corticosterone, the main glucocorticoid in rodents (Gong et al. 2015), leptin, a 16 kDa protein secreted by adipocytes, which has roles in controlling energy balance as well as regulating responses to stress (Haleem 2014), and glucose, since cortisol counters insulin by promoting higher blood sugar and stimulating gluconeogenesis, for synthesizing glucose (Maniam et al. 2014). Biomarkers of HPA activity could not fully explain the effects observed in the transgenic rats. In the case of corticosterone, acute stress (foot shock) induced increased plasma levels, but in both transgenic and Wt rats, to serve as a good biological validator of the stress paradigm the rats were subjected to. Exposure to the stressor did not alter levels of either leptin or glucose, with levels of these markers of HPA activity that were also independent of ChAT::Cre− genotype. It is possible that sex differences may exist in both the endocrine, cognitive behavioral and stress responses shown by the ChAT::Cre+ rats as a result of increased gene copies of *VAChT*, which translated to overexpressed VAChT protein levels. Future work should further investigate for such potential sex-specific differences.

The current work differentially analyzed VAChT protein and mRNA levels between the transgenic ChAT::Cre+ and non-transgenic ChAT::Cre− rats to PFC brain tissue samples. As a brain region that contains dense cholinergic innervation and outputs, the PFC was deemed to play multiple functional roles that particularly relate to the cognitive processes that the battery of behavioral tests used in the present study were designed for (Wallis 2007; Euston et al. 2012, Funahashi 2013). This includes strong arguments that have been made that ACh signaling in the PFC is strategically uniquely positioned at both the start and terminal end of the attentional loop (Sarter et al. 2001; Parikh et al. 2007). Through a series of tests, we also undertook anxiety profiling in ChAT::Cre+ versus ChAT::Cre− rats. Again, several studies implicated the PFC in anxiety-related behavior, with the PFC that was shown to exert integral control (via cholinergic reciprocal connections) over the limbic system (Kim and Whalen 2009). The strength of the connectivity between the PFC and the amygdala has further been correlated with anxiety-like behavior shown in both the OF and EPM (Wei et al. 2010; Delpech et al. 2016). The OF and EPM paradigms inherently also evaluate aspects of motor control, including locomotion and explorative behavior. A comprehensive body of literature implies an important role for forebrain ACh in controlling these motor-related aspects, as it exerts top-down control over several motor areas via reciprocal connections with the PFC (Asaad et al. 2000; Miller and Cohen 2001).

However, cholinergic neurotransmission throughout the neocortex, but also the hippocampus, amygdala and PPN can regulate arousal, learning and attention as well as aspects of emotional processing (Inglis et al. 2000, 2001). Hence, future work should aim to determine whether the presently reported molecular findings pertaining to the PFC is specific to this cholinergic brain region, or whether a similar VAChT protein and mRNA level expression pattern is evident for other brain regions containing cholinergic projection neurons in ChAT::Cre+ compared to ChAT::Cre− rats.

Taken together, the current results reveal that the ChAT::Cre rat line overexpresses ‘extra’ copies of a gene that functionally control cholinergic signaling. A similar effect where the BAC transgene also introduces extra copies of adjacent genes to the genome is predicted to occur in another popular transgenic rat line, namely TH::Cre+ rats (Witten et al. 2011), with such rats expressing Cre recombinase in tyrosine hydroxylase (TH) neurons. As TH acts as the rate-limiting enzyme of catecholamine biosynthesis, this rat line has, therefore, been engineered to express Cre in catecholaminergic neurons, including dopaminergic ones, that are affected progressively during neurodegenerative diseases such as PD, and has, therefore, become particularly useful for researching the pathological mechanisms concerning PD. Hence, future efforts for developing novel transgenic rat systems should utilize strategies for developing cleaner transgenic lines by eliminating unwanted genes from modified BAC DNA that could complicate phenotypic analysis.

The increasing use of genetic models, which include transgenic knockout and inbred strains of rodents has highlighted the importance of detailed characterization at behavioral and molecular-genetic levels of such rodents being used for pursuing research questions. A milestone was reached with the development of Cre/lox recombination techniques to enable conditional gene targeting in rodents. The technology utilizes *Cre* recombinase that excises a gene segment flanked by pairs of specific 34-bp DNA sequences termed LoxP recognition sequences (McLellan et al. 2017). Use of cell-specific drivers of Cre restricts recombination exclusively to cells where the driver gene is expressed, hence generating conditional knockout or knockin animals. However, the desired tissue specificity may be difficult to apply using Cre transgenic lines, with mosaic expression, leakiness of the promoter, Cre-induced toxicity, or Cre non-specificity that have been reported, all of which could confound the original purpose of the experiments. With this regard, several published reports have highlighted potential limitations posed by the use of ChAT::Cre+ mouse lines in behavioral research (e.g., Crittenden et al. 2014; Chen et al. 2018). Hence, our study provides evidence, for the first time, to strongly suggest that caution should prevail when interpreting data derived from ChAT::Cre+ rats, for functionally modulating cholinergic signaling, particularly in the utilization of cre/lox technology for evaluating the in vivo effects of the *ChAT* gene on performance in cognitive tasks.

## Abbreviations

ACh: Acetylcholine
AChE: Acetylcholinesterase
AD: Alzheimer’s disease
ADHD: Attention deficit/hyperactivity disorder
BAC: Bacterial artificial chromosome
BSA: Bovine serum albumin
5-CSRTT: Five-choice serial reaction time task
ChR2: Channelrhodopsin-2
ChAT: Choline acetyltransferase
CFCT: Contextual fear conditioning task
DREADD: Designer receptors exclusively activated by designer drugs
DAT: Dopamine transporter
EPM: Elevated plus maze
eYFP: Enhanced yellow fluorescent protein
EtOH: Ethanol
CHT1: High-affinity choline transporter
HSD: Honestly significant difference
HPA: Hypothalamic–pituitary–adrenal
ITI: Intertrial interval
LSD: Least significant difference
LDB: Light/dark box
LH: Limited hold
NS: Non-significant
NBMc: Nucleus basalis magnocellularis
NBM: Nucleus basalis of Meynert
OF: Open field
PD: Parkinson’s disease
PPN: Pedunculopontine nucleus
PCR: Polymerase chain reaction
PVDF: Polyvinylidene difluoride
PFC: Prefrontal cortex
qPCR: Quantitative PCR
RIPA: Radioimmunoprecipitation assay
RT-PCR: Real-time PCR
RRID: Research resource identifier
RT: Room temperature
SLC5A7: Solute carrier family 5 member 7
SD: Stimulus duration
TBS-T: Tris-buffered saline-Tween 20
TH: Tyrosine hydroxylase
VAChT: Vesicular acetylcholine transporter
Wt.: Wild-type
